# Leveraging Electrical Network Models for Solving Fick’s Second Law of Diffusion in Membrane Gas Permeation

**DOI:** 10.3390/membranes16050165

**Published:** 2026-05-01

**Authors:** Zheng Cao, Boguslaw Kruczek, Jules Thibault

**Affiliations:** Department of Chemical and Biological Engineering, University of Ottawa, Ottawa, ON K1N 6N5, Canada; zcao088@uottawa.ca (Z.C.); bkruczek@uottawa.ca (B.K.)

**Keywords:** electrical analogy, membrane gas permeation, Fick’s law of diffusion, finite differences, MMMs

## Abstract

The permeation of gases through membranes is a fundamental process with wide-ranging applications, from gas separation and fuel cell technology to respiratory physiology. Governed by Fick’s second law of diffusion, the mathematical modelling of such transport processes often becomes analytically and computationally challenging, especially in heterogeneous, mixed matrix, or multilayered systems. To navigate these complexities, this study revisits and expands upon the use of electrical analogies as an intuitive and powerful modelling approach rooted in mid-20th-century analog computing. By leveraging the mathematical equivalence between diffusion and electrical conduction, we construct an equivalent electrical network that mirrors the transient behaviour of gas permeation across membranes. In this framework, concentration gradients are represented as voltage differences, diffusive fluxes as electrical currents, and diffusional resistances as circuit resistances. While traditional applications of electrical analogies have largely focused on steady-state phenomena, our approach enables dynamic analysis, offering conceptual clarity and computational efficiency. This methodology not only simplifies the solution of Fick’s second law but also reinforces the enduring relevance of analogical thinking in modern engineering practice. Comparative results demonstrate that the equivalent electrical circuit closely aligns with both analytical and finite difference solutions, validating its effectiveness and accuracy.

## 1. Introduction

The permeation of gases through membranes is a fundamental transport process relevant to a wide range of scientific and engineering applications, including gas separation, fuel cell operation, and respiratory physiology. In many such systems, transport is governed by Fick’s second law of diffusion, which characterizes the temporal evolution of concentration profiles within a diffusing medium. Analytical and numerical solutions to this governing equation can become highly complex, particularly in heterogeneous or multilayered membrane systems. In addition, experimental determination of the associated transport parameters is often challenging due to the intricacies of these systems. To overcome these difficulties, researchers have long employed analogical methods as both conceptual and practical tools for modelling transport phenomena. Analogies provide a unifying framework that links disparate engineering principles and facilitates the interpretation of underlying physical mechanisms across disciplines. A prominent example is the Chilton–Colburn *j*-factor analogy, which formalizes the similarity among momentum, heat, and mass transfer in fluid systems, especially under turbulent flow conditions [1,2]. Such analogies are particularly valuable because they enable the estimation of difficult-to-measure quantities, such as mass transfer coefficients, using more readily obtained heat or momentum transfer data [3,4]. Numerous studies have demonstrated the utility of this approach, employing mass transfer experiments to infer heat transfer parameters and vice versa by exploiting the mathematical parallels between the two processes. Heat and mass transfer frequently share analogous governing laws (e.g., Fourier’s law for heat conduction and Fick’s law for diffusion), allowing one phenomenon to serve as a surrogate for the other in experimental investigations. For instance, mass transfer experiments (e.g., using naphthalene sublimation or electrochemical methods) often provide more controlled and accessible measurements than direct heat transfer experiments [5,6,7,8].

Historically, the electrical analogy has been one of the most widely employed conceptual tools in engineering. Until approximately 1980, analog computers remained prevalent for simulating complex process dynamics in both engineering practice and scientific research. These devices relied on electrical circuits to represent differential equations, enabling users to visualize and manipulate system behaviour directly. By providing an interactive, physically grounded means of modelling, analog computers rendered abstract mathematical formulations more accessible. They were regarded as technological milestones of mid-twentieth-century engineering, particularly for the simulation of fluid flow, mechanical vibrations, chemical reaction networks, and control systems prior to the widespread adoption of digital computation. Engineers could tweak parameters in real time and immediately observe the resulting system response. With the advent of digital computing, analog machines were gradually phased out and now largely reside in museums [9,10]. Despite the obsolescence of analog computers themselves, the electrical analogy continues to play a central role in engineering education and practice, offering an intuitive framework for understanding complex physical phenomena. It remains successfully applied across a broad spectrum of engineering domains, including thermal conduction and radiation [11], process control [12], system dynamics [13], and acoustics [14].

Electrical analogies leverage the mathematical equivalence between diffusion and electrical conduction, enabling mass transport phenomena to be represented through analogous electrical circuits. Within this framework, concentration gradients are treated as voltage differences, diffusive fluxes as electrical currents, and diffusional resistances as electrical resistances. This correspondence enables the use of established circuit analysis methods to address diffusion problems, providing both conceptual clarity and computational efficiency. Equivalent electrical circuits have been used for decades to elucidate heat and mass transfer mechanisms. For example, Yagi and Kunii [15] used an electrical circuit representation to model heat transfer in a packed bed, while Kim and Yeo [16] applied a similar approach to analyze the dynamic behaviour of thermal bridges in building envelopes. Electrical analogies have also been adopted in fluid flow studies [17]. More recently, Aït-Idir et al. [18] used an equivalent electrical circuit to investigate oxygen transport in polymer electrolyte membrane fuel cells.

Most studies employing equivalent electrical circuits have used them primarily as illustrative tools for interpreting physical phenomena under steady-state or relatively simple transient conditions. This is also the case for membrane permeation. For example, Fouda et al. [19] proposed a resistance model, analogous to a Wheatstone bridge, for gas transport in composite membranes, specifically introducing a parallel flow model to better explain experimental data for high-permeation gases like hydrogen. He et al. [20] used a resistance model for gas permeation in a composite membrane, which was divided into five regions with their respective thickness and resistance. In this work, we extend the electrical analogy framework to model transient gas permeation through membranes. By formulating an equivalent electrical circuit that faithfully represents the underlying diffusion process, we show that Fick’s second law of diffusion can be solved with enhanced clarity and reduced computational complexity. This development advances the role of electrical analogies from conceptual aids to practical, dynamic modelling methodologies for diffusion-dominated systems.

## 2. Methodology

This section presents the derivation of the mathematical framework employed to model species permeation through both neat and mixed-matrix membranes (MMMs). Two complementary methodologies are considered: (i) the numerical solution of Fick’s second law of diffusion using a finite-difference scheme, which serves as a reference/benchmark for assessing the accuracy of the electrical-circuit approach, and (ii) the formulation of one-dimensional and three-dimensional equivalent electrical circuit models that replicate the same fundamental transport mechanisms.

### 2.1. Gas Permeation Through a Membrane

**Figure 1:**
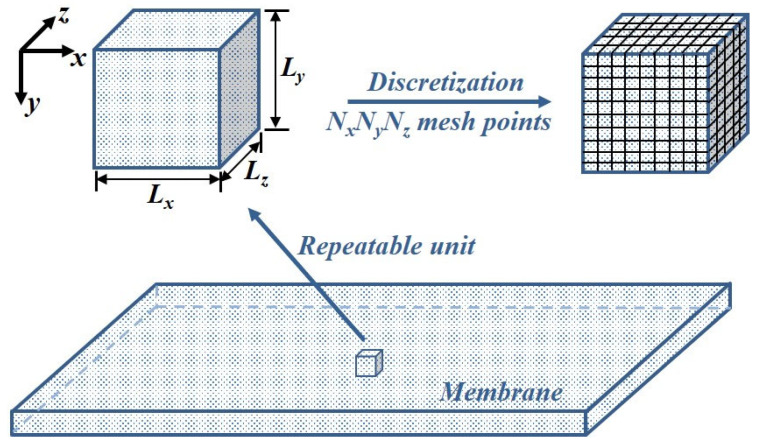
Illustration of a three-dimensional representative repeatable unit of a membrane of dimension *L_x_*, *L_y_* and *L_z_*, and its discretization necessary to solve numerically for the temporal and spatial concentration within the membrane.

To characterize the spatiotemporal evolution of species concentration within a membrane during a time-lag experiment, Fick’s second law of diffusion must be solved. For a homogeneous membrane, the absence of a longitudinal concentration gradient reduces the problem to a one-dimensional (1D) formulation in the permeation direction under ideal boundary conditions. In this simplified case, an analytical solution for both concentration and flux as functions of time and position can be readily obtained using the method of separation of variables. In contrast, heterogeneous or anisotropic membranes require numerical solution of the full three-dimensional (3D) form of Fick’s second law. This situation arises in composite and MMMs, as well as in membranes exhibiting spatially varying permeability. In the present work, we address the case of a MMM comprising a monolayer of small cuboid particles uniformly embedded within a polymer matrix. The particles possess diffusivity and solubility values distinct from those of the polymer phase, although both phases are assumed isotropic. To compute the transient and spatial concentration fields, a representative repeatable unit is defined, consisting of a single particle centred within a surrounding polymer domain. The unit is constructed such that the particle volume fraction matches that of the overall membrane. All repeatable units are identical, and their periodic arrangement constitutes the full MMM structure. Prior work has demonstrated that the effective permeability of a permeating species is identical for a single repeatable unit and for the entire membrane [21]. Consequently, the numerical solution of Fick’s second law for a single repeatable unit is sufficient to describe transport through the full MMM. Figure 1 presents a schematic of the repeatable unit and its discretization in Cartesian coordinates into (*N_x_* × *N_y_* × *N_z_*) mesh points.

The governing equation for Fick’s second law of diffusion in the MMM relates the temporal rate of change in species concentration to the divergence of the diffusive flux, as expressed in Equation (1).

(1) $$\frac{\partial c}{\partial t}=D(x,y,z)\nabla^{2}c=D(x,y,z)\left(\frac{\partial^{2}c}{\partial x^{2}}+\frac{\partial^{2}c}{\partial y^{2}}+\frac{\partial^{2}c}{\partial z^{2}}\right)$$

**Figure 2:**
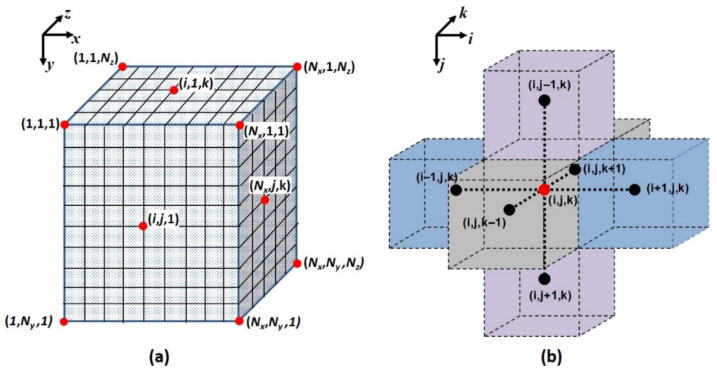
Nomenclature of (**a**) the discretization of a repeatable unit into (*N_x_* − 1) × (*N_y_* − 1) × (*N_z_* − 1) mesh elements and (**b**) of an interior mesh point with its six neighbouring mesh points. *x*, *y* and *z* directions are represented by indices *i*, *j* and *k*, respectively.

In this equation, *c* denotes the concentration of permeating species at a position (*x*,*y*,*z*) and time (*t*). *D*(*x*,*y*,*z*) indicates that the diffusivity within the MMM may vary spatially. Figure 2a presents the notation of the discretization scheme for the repeatable unit, and Figure 2b depicts an interior mesh element (*i*,*j*,*k*), together with its six neighbouring mesh elements, which are used to discretize Equation (1). An ideal interface between the polymer matrix and the embedded particle is assumed; that is, no interfacial voids, rigidified polymer layers, or pore-blockage regions are considered. Consequently, the system is treated as comprising two distinct isotropic phases, the polymer and the filler particle, each characterized by its own diffusion and solubility coefficients. Prior to the step change in pressure of a time-lag permeation experiment, the membrane is assumed to be fully degassed, leading to the initial condition specified in Equation (2).

(2) $$\begin{array}{cc} IC: & C_{x,y,z}^{t<0}=0 \end{array}\;\;\;\;\forall x,y,z$$

The boundary conditions applied at the membrane surfaces consist of a fixed gas concentration *C*_0_ at the feed–membrane interface and a perfect vacuum at the permeate–membrane interface. These boundary conditions are expressed in Equations (3) and (4).

(3) $$BC_{1}:\;\;\;c_{x,y=0,z}=SC_{0}\;\mathrm{for}\;\forall x,z$$

(4) $$BC_{2}:\;\;\;c_{x,y=L_{y},z}=0\;\mathrm{for}\;\forall x,z$$

Because the membrane consists of a large number of juxtaposed repeatable units, symmetry conditions apply along the four lateral surfaces of each unit. These symmetry conditions constitute the remaining four boundary conditions of Equation (1), as expressed in Equation (5).

(5) $$BC_{3-6}:{\left.\frac{\partial c}{\partial x}\right|}_{x\;=\;0}={\left.\frac{\partial c}{\partial x}\right|}_{x\;=\;L_{x}}={\left.\frac{\partial c}{\partial z}\right|}_{z\;=\;0}={\left.\frac{\partial c}{\partial z}\right|}_{z\;=\;L_{z}}=0$$

As illustrated in Figure 2b, six mass fluxes occur across the six interfacial surfaces separating the central mesh element from its neighbouring elements. Adjacent mesh elements may possess different transport properties, corresponding either to the polymer phase (*D_P_*, *S_P_*) or the filler particle (*D_F_*, *S_F_*). Wu et al. [22] examined this situation, where neighbouring mesh elements may differ in their transport properties, and derived a discretized finite-difference formulation applicable to all interior mesh points within the MMM (Equation (6)). This formulation represents the sum of the six interfacial fluxes, each evaluated using Fick’s first law of diffusion. The six diffusion coefficients $\left(D_{i,j,k}^{LX},D_{i,j,k}^{RX},D_{i,j,k}^{LY},D_{i,j,k}^{RY},D_{i,j,k}^{LZ},\;\mathrm{and}\;D_{i,j,k}^{RZ}\right)$ appearing in Equation (6) are effective diffusivities that account for both the diffusivity and solubility of the materials on either side of each interface. For example, the effective coefficient $D_{i,j,k}^{LX}$ is defined in Equation (7), and the remaining five coefficients follow analogously. When all mesh elements consist of the same material, the solubility ratio reduces to unity, and the effective diffusivity simplifies to the intrinsic material diffusivity. Appendix A briefly discusses how the transport properties of the membrane and the filler can be obtained.

(6) $$c_{i,j,k}^{t+\Delta t}=c_{i,j,k}^{t}+\Delta t\left[\begin{array}{l} -D_{i,j,k}^{LX}\frac{\left(c_{i,j,k}^{t}-\frac{S_{i,j,k}}{S_{i-1,j,k}}c_{i-1,j,k}^{t}\right)}{\Delta x^{2}}+D_{i,j,k}^{RX}\frac{\left(\frac{S_{i,j,k}}{S_{i+1,j,k}}c_{i+1,j,k}^{t}-c_{i,j,k}^{t}\right)}{\Delta x^{2}}\\ -D_{i,j,k}^{LY}\frac{\left(c_{i,j,k}^{t}-\frac{S_{i,j,k}}{S_{i,j-1,k}}c_{i,j-1,k}^{t}\right)}{\Delta y^{2}}+D_{i,j,k}^{RY}\frac{\left(\frac{S_{i,j,k}}{S_{i,j+1,k}}c_{i,j+1,k}^{t}-c_{i,j,k}^{t}\right)}{\Delta y^{2}}\\ -D_{i,j,k}^{LZ}\frac{\left(c_{i,j,k}^{t}-\frac{S_{i,j,k}}{S_{i,j,k-1}}c_{i,j,k-1}^{t}\right)}{\Delta z^{2}}+D_{i,j,k}^{RZ}\frac{\left(\frac{S_{i,j,k}}{S_{i,j,k+1}}c_{i,j,k+1}^{t}-c_{i,j,k}^{t}\right)}{\Delta z^{2}} \end{array}\right]$$

(7) $$\frac{1}{D_{i,j,k}^{LX}}=\frac{S_{i}}{S_{i-1}}\frac{1}{2D_{i-1,j,k}}+\frac{1}{2D_{i,j,k}}$$

### 2.2. Electrical Equivalent for a Neat Membrane (One-Dimensional)

**Figure 3:**
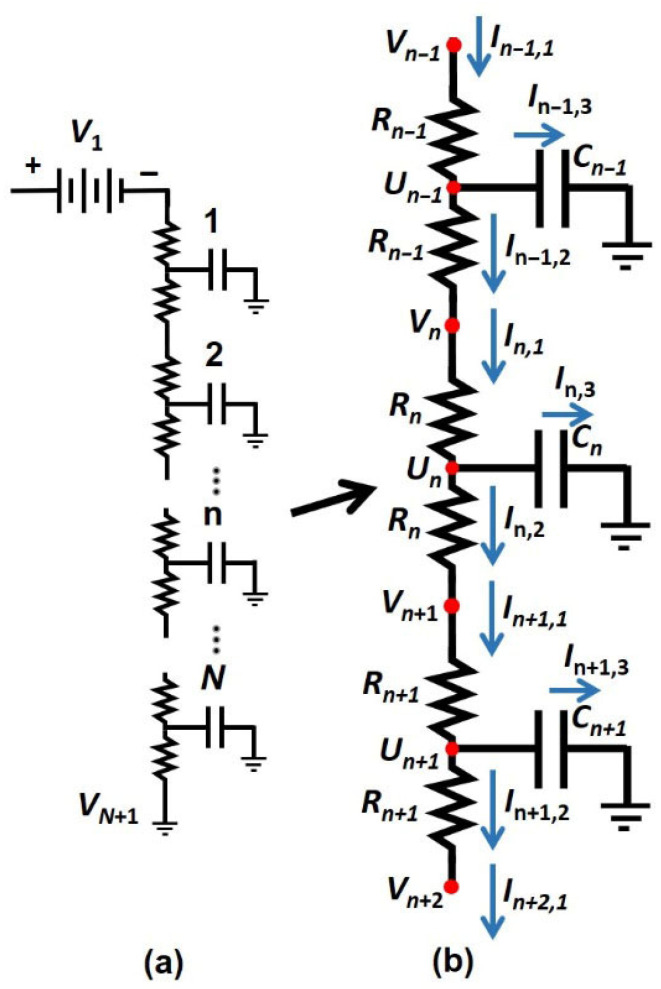
An equivalent electrical circuit representing a neat membrane under the one-dimensional formulation of Fick’s second law of diffusion: (**a**) the membrane is discretized into *N* elementary circuit elements, and (**b**) three consecutive elementary elements with nomenclature used.

The mathematical analogy between unsteady-state heat and mass transfer and Ohm’s law enables the use of an equivalent dynamic electrical circuit to model transport phenomena. When appropriate electrical analogies of the mass-transfer parameters are applied, the iterative solution of the circuit shown in Figure 3 can be used to determine the permeation of a chemical species through a homogeneous membrane. In this case, we are aiming to solve the one-dimensional (1D) Fick’s second law of diffusion. To simulate the evolution of permeation from the initial (ground) state to the steady state, the electrical circuit in Figure 3 is solved according to the following procedure.

(a) The domain of solution is discretized into *N* elementary electrical circuits, as illustrated in Figure 3. Each elementary circuit comprises two resistances *R_n_*, and one capacitor *C_n_*, representing the total resistance and capacitance, respectively, of the *n^th^* circuit elementary. In the mass transfer analogy, the total resistance 2*R_n_* of an elementary circuit corresponds to (Δ*y*/*AD_n_S_n_*) as shown in Table 1, where Δ*y* is the thickness of the associated membrane layer (Δ*y* = *L_y_*/*N*), *A* is the membrane area, *D_n_* is the diffusivity of the nth layer, and *S_n_* is its solubility. The charge accumulated in the capacitor is proportional to the local concentration of the permeating species, with the capacitance given by (*A*Δ*yS_n_*).

(b) All voltages (*V_n_* and *U_n_*), currents (*I_n_*_,1_, *I_n_*_,2_, and *I_n_*_,3_), and accumulated charge (*Q_n_*) of each elementary circuit are initially set to zero. At time *t* = 0, the surface voltage *V*_1_, the input voltage of the first elementary circuit, is assigned a value corresponding to the equilibrium surface concentration at the membrane interface, typically *C*_0_*S*_1_ or *p*_0_*S*_1_/*RT*. Simultaneously, the voltage at the downstream side of the membrane, represented by the output voltage *V_N+_*_1_ of the *Nth* elementary circuit, is set to zero, reflecting the assumption of a perfect vacuum at the permeate side. For the one-dimensional case of a homogeneous membrane, this initial condition, together with the two boundary conditions, fully defines the problem to be solved.

(c) At each integration time step Δ*t*, the currents in the three branches of every elementary circuit are evaluated using Ohm’s law. These currents are then used to compute the incremental increase in the accumulated charge at each iteration, as described by Equations (8)–(11).

(8) $$I_{n,1}=\frac{V_{n}-U_{n}}{R_{n}}$$

(9) $$I_{n,2}=\frac{U_{n}-U_{n+1}}{R_{n}+R_{n+1}}$$

(10) $$I_{n,3}=I_{n,1}-I_{n,2}$$

(11) $$\frac{dQ_{n}}{dt}=I_{n,3}^{t}\;\Rightarrow \;Q_{n}^{t+\Delta t}=Q_{n}^{t}+I_{n,3}^{t}\Delta t$$

(d) The time-dependent increase in the accumulated charge within the capacitor yields the updated value of the central voltage *U_n_* for each elementary electrical circuit, as determined by Equation (12).

(12) $$U_{n}^{t+\Delta t}=\frac{Q_{n}^{t+\Delta t}}{C_{n}}$$

Using the updated values of the voltage *U_n_*, the input voltage *V_n_* for each elementary circuit is subsequently computed from Equation (13), while the surface voltage *V*_1_ remains fixed.

(13) $$V_{n}^{t+\Delta t}=\frac{R_{n-1}R_{n}}{R_{n-1}+R_{n}}\left[\frac{U_{n-1}^{t+\Delta t}}{R_{n-1}}+\frac{U_{n}^{t+\Delta t}}{R_{n}}\right]\;\;\;\;for\;n=2\;to\;N$$

This procedure is iterated over a large number of integration time steps Δ*t* until a steady state is reached. The currents *I*_1,1_ and *I_N_*_,2_ represent the instantaneous fluxes at the upstream and downstream membrane surfaces, respectively.

### 2.3. Electrical Equivalent for a Three-Dimensional (3D) Membrane

**Figure 4:**
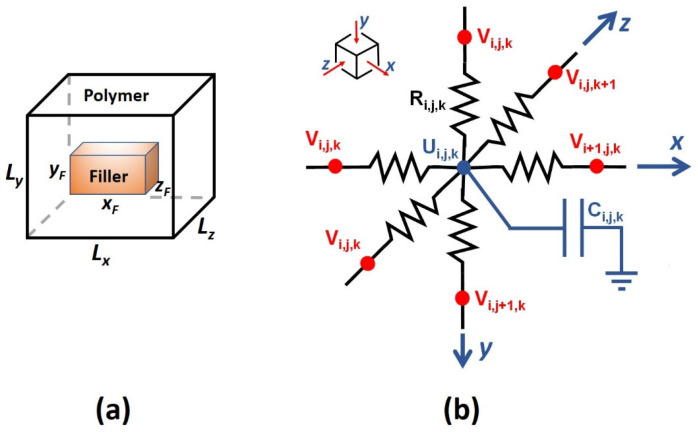
(**a**) Schematic of a MMM with a filler located at the center of an elementary unit; (**b**) the electrical circuit configuration of a discrete volume necessary to solve the three-dimensional solution of Fick’s second law of diffusion.

The one-dimensional (1D) equivalent electrical circuit used to model the transient permeation of a chemical species through a neat, homogeneous membrane can be readily extended to a three-dimensional electrical network. This extension enables the solution of Fick’s second law of diffusion for MMMs, which serve as the three-dimensional case study in this investigation (Figure 4a). For the illustrative example considered here, the MMM is assumed to be ideal: all solid filler particles possess identical shape, size, and orientation, and are uniformly dispersed throughout the polymer matrix. The polymer–particle interface is assumed ideal, and the permeability and solubility coefficients of both phases are taken as constant. The two-phase MMM is partitioned, as with the finite difference discretization, into a series of elementary units. Each unit consists of a polymer matrix containing a centrally positioned solid particle, as schematically depicted in Figure 4a. The particle volume fraction within an elementary unit is identical to that of the overall membrane. In Cartesian coordinates, the three-dimensional elementary unit of dimensions *L_x_* × *L_y_* × *L_z_* is discretized into a grid comprising *N_x_* × *N_y_* × *N_z_* discrete volumes. The centrally located filler particle, with dimensions *x_F_* × *y_F_* × *z_F_*, is characterized by a diffusivity *D_F_* and a solubility *S_F_*, while the surrounding polymer phase possesses diffusivity *D_P_* and solubility *S_P_*.

Each 3D discrete volume within the elementary unit of the membrane is represented by the electric circuit shown in Figure 4b. Six branches, two along each spatial dimension, are connected to the central node of the discrete volume. All six resistances *R_i,j,k_* associated with a given volume (*i,j,k*) are identical within that volume; their values may differ between volumes. For example, the resistances of a polymer region differ from those of a filler particle. The capacitance *C_i,j,k_* may also vary from one discrete volume to another, depending on the local volume Δ*x*Δ*y*Δ*z* and solubility *S_i,j,k_*. The resistance and capacitance of each discrete volume are defined using the mass-transfer analogies listed in Table 1. Each interior discrete volume is updated according to the following procedure. At every integration time step Δ*t*, the currents in the six branches of each discrete volume are computed using Ohm’s law and subsequently used to determine the excess current *I_i,j,k,C_* entering the capacitor, as given by Equation (14). Subscripts 1 and 2 denote the currents entering and leaving through the two branches in the *x*-direction, respectively. Similarly, subscripts 3 and 4 correspond to the *y*-direction, and 5 and 6 correspond to the *z*-direction.

(14) $$\begin{array}{l} I_{i,j,k,C}=I_{i,j,k,1}-I_{i,j,k,2}+I_{i,j,k,3}-I_{i,j,k,4}+I_{i,j,k,5}-I_{i,j,k,6}\\ \;\;\;\;\;\;\;\;=\frac{U_{i,j,k}-U_{i-1,j,k}}{R_{i-1,j,k}+R_{i,j,k}}-\frac{U_{i+1,j,k}-U_{i,j,k}}{R_{i,j,k}+R_{i+1,j,k}}+\frac{U_{i,j,k}-U_{i,j-1,k}}{R_{i,j-1,k}+R_{i,j,k}}-\frac{U_{i,j+1,k}-U_{i,j,k}}{R_{i,j,k}+R_{i,j+1,k}}\\ \;\;\;\;\;\;\;\;+\frac{U_{i,j,k}-U_{i,j,k-1}}{R_{i,j,k-1}+R_{i,j,k}}-\frac{U_{i,j,k+1}-U_{i,j,k}}{R_{i,j,k}+R_{i,j,k+1}} \end{array}$$

The excess current *I_i,j,k,C_* associated with the discrete volume (*i*,*j*,*k*), represents the portion of the total current that flows into the capacitor and is therefore used to compute the incremental increase in the accumulated charge at each iteration, as described by Equation (15).

(15) $$\frac{dQ_{i.j.k}}{dt}=I_{i,j,k,C}^{t}\;\;\;\Rightarrow \;\;\;Q_{i,j,k}^{t+\Delta t}=Q_{i,j,k}^{t}+I_{i,j,k,C}^{t}\Delta t$$

Consistent with Equations (12) and (13) for the 1D electric circuit, the voltage at the center of each discrete volume *U_i,j,k,_* is first updated, after which the interfacial voltages *V_i,j,k_* between adjacent volumes are computed. These updated voltages enable the next iteration of the solution procedure, which is repeated until a steady state is attained.

Equation (14) applies to all interior discrete volumes. To obtain a complete solution, both initial and boundary conditions must be specified. Prior to the step change in concentration or pressure at the feed side of the membrane at time *t* = 0, the membrane is assumed to be at a uniform zero voltage (i.e., zero concentration), as expressed in (Equation (16)). Six boundary conditions are required, one for each face of the repeatable elementary unit, to fully define the problem. Equation (17) specifies the boundary conditions on the upstream and downstream surfaces of the membrane, with permeation occurring along the *y*-axis. At the onset of permeation, a step change in gas pressure or concentration is imposed at the upstream surface (*y* = 0), while the downstream surface of the membrane (*y* = *L_y_*) is maintained under perfect vacuum. The corresponding concentrations at these membrane surfaces are given by the product of the adjacent gas pressure or concentration and the polymer solubility. Because all elementary units are identical, symmetry conditions apply at the remaining four faces, as expressed in Equation (18) for *BC*_3–6_. These periodic boundary conditions reflect the fact that the MMM consists of a large array of identical elementary units.

(16) $$\begin{array}{c} IC:\;\;\;V_{i,j,k}^{t<0}=U_{i,j,k}^{t<0}=0 \end{array}\;\;\;\forall i,j,k$$

(17) $$\begin{array}{l} BC_{1}:\;V_{i,j\;=\;1,k}=\frac{p_{0}}{RT}S_{i,1,k}{=c}_{0}S_{i,1,k}\;\;\;\forall i,k\\ BC_{2}:\;V_{i,j\;=\;N_{y}+1,k}=0\;\;\;\;\;\;\;\;\;\;\;\;\;\;\;\;\;\;\;\;\;\;\;\;\;\forall i,k \end{array}$$

(18) $$\begin{array}{l} BC_{3}:\;I_{i=1,j,k,1}=0\;\;\;\;\;\;\;\;\;\;\;\;\forall j,k\\ BC_{4}:\;I_{i=N_{x},j,k,2}=0\;\;\;\;\;\;\;\;\;\;\;\forall j,k\\ BC_{5}:\;I_{i,j,k=1,5}=0\;\;\;\;\;\;\;\;\;\;\;\;\forall i,j\\ BC_{6}:\;I_{i,j,k=N_{z},6}=0\;\;\;\;\;\;\;\;\;\;\;\forall i,j \end{array}$$

## 3. Results and Discussion

### 3.1. Neat Membrane (1D Solution)

**Figure 5:**
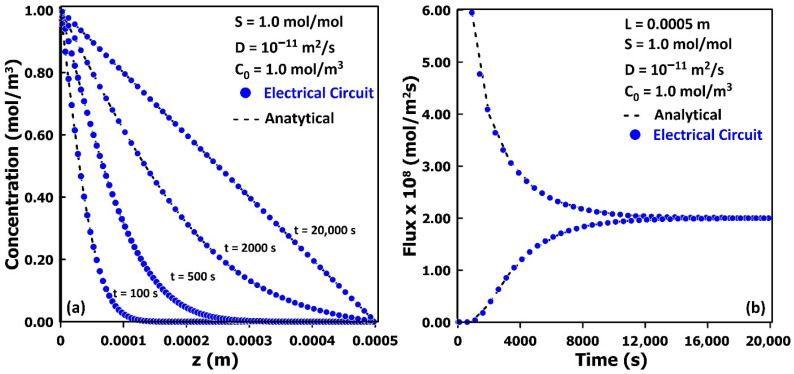
Comparison of the analytical solution and the numerical solution obtained using the electrical circuit for (**a**) the concentration profile across the membrane at four different times, and (**b**) the upstream and downstream fluxes as a function of time.

To assess the accuracy of the equivalent electrical circuit in Figure 3 for simulating the transient permeation of a chemical species through a neat polymeric membrane, the numerical results are compared with the analytical solution given in Equation (19) and are presented in Figure 5a. When the resistances and capacitances of the circuit are assigned according to their mass-transfer analogies (Table 1), the time-dependent voltage profile obtained from the electrical network directly yields the corresponding concentration profile across the membrane, without requiring any back-substitution. As shown in Figure 5a, the predicted concentration profiles closely match the analytical solution throughout the entire permeation process.

Figure 5b shows the instantaneous inlet and outlet fluxes obtained from the simulation of gas permeation through a non-porous membrane using the electrical-circuit analogy. The fluxes at the feed and permeate sides are computed directly from the sums of the input and output current densities in the electrical network. At the onset of permeation, the upstream flux is initially high due to the large voltage (concentration) difference imposed across the membrane. As the voltage within the membrane gradually increases, the inlet flux decreases until a constant permeation rate is established. In contrast, the outlet flux remains zero until molecules originating from the higher upstream concentration traverse the membrane and begin to appear at the downstream side, eventually reaching a constant value equal to the steady-state permeation rate. The dotted curve in Figure 5b corresponds to the instantaneous inlet and outlet fluxes predicted by the analytical solution given in Equations (20) and (21). The close agreement between the analytical and numerical results demonstrates that the equivalent electrical-circuit model, when parameterized according to the mass transfer analogies in Table 1, accurately captures both the transient concentration profiles and the associated inlet and outlet fluxes.

(19) $$C(y,t)=C_{0}S\left(1-\frac{y}{L_{y}}\right)-\left(\frac{2C_{o}S}{\pi }\right)\;\sum_{n=1}^{\infty }\frac{1}{n}\sin\left(\frac{n\pi y}{L_{y}}\right)e^{-\left(\frac{Dn^{2}\pi^{2}t}{L_{y}^{2}}\right)}$$

(20) $$J_{u}=J(0,t)=\frac{DC_{0}S}{L_{y}}+\left(\frac{2DC_{o}S}{L_{y}}\right)\;\sum_{n=1}^{\infty }e^{-\left(\frac{Dn^{2}\pi^{2}t}{L_{y}^{2}}\right)}$$

(21) $$J_{d}=J(L_{y},t)=\frac{DC_{0}S}{L_{y}}+\left(\frac{2DC_{o}S}{L_{y}}\right)\;\sum_{n=1}^{\infty }\cos(n\pi )\;e^{-\;\left(\frac{Dn^{2}\pi^{2}t}{L_{y}^{2}}\right)}$$

### 3.2. Mixed Matrix Membrane (3D Solution)

The applicability of the electric-circuit analogy for solving Fick’s second law of diffusion in three-dimensional (3D) systems is evaluated by examining gas permeation through an MMM. In such membranes, solid filler particles of various geometries are embedded within a continuous polymer matrix. These fillers play a critical role in enhancing membrane performance beyond what is achievable by the polymer alone. Materials such as zeolites, metal–organic frameworks (MOFs), and silica can function as molecular sieves, selectively permitting the passage of certain species while hindering others, thereby improving separation efficiency. In addition, some fillers introduce alternative diffusion pathways to alter the tortuosity of transport, consequently modifying the overall membrane permeability [23]. In this section, the electric circuit depicted in Figure 4 is employed to predict the permeability of a MMM functioning as a barrier material, where the filler possesses a relatively low permeability (*P_F_* = 10^−18^ m^2^/s) compared with that of the polymer matrix (*P_p_* = 10^−12^ m^2^/s). Because no analytical three-dimensional solution exists for MMMs, the same problem was independently solved using a finite difference approach. The resulting comparison is used to assess the accuracy of the electrical-circuit equivalency.

**Figure 6:**
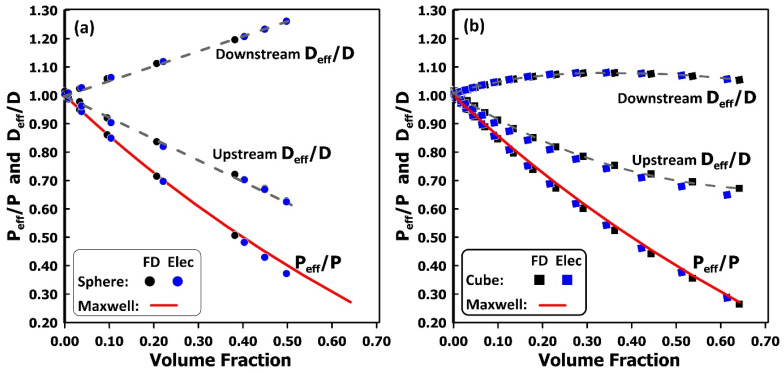
Comparison of the electrical circuit analogy, the finite difference method, and the Maxwell equation for estimating the relative permeability of a MMM containing (**a**) spherical and (**b**) cubic fillers as a function of the filler volume fraction. Also shown are the relative diffusivities of the MMM obtained from the upstream and downstream time-lag methods. Dash lines are trend lines.

A series of simulations was conducted using spherical and cubic filler particles of various sizes. The resulting predictions are presented in Figure 6, where the relative membrane permeability is plotted as a function of the filler volume fraction. The relative permeability is defined as the ratio of the effective permeability of the MMM to that of the neat polymer membrane (*P_eff_*/*P_p_*). Figure 6a compares the relative permeability of the MMM containing spherical fillers as obtained from the equivalent electrical-network model, the finite difference method, and the classical Maxwell equation [24]. The Maxwell equation, given in Equation (22), was originally derived to estimate the effective permeability of a dispersion of spherical inclusions within a continuous matrix of permeabilities *P_F_* and *P_P_*, respectively. Although typically recommended for filler volume fractions up to approximately 0.25, the results indicate that the Maxwell equation maintains reasonably good accuracy even at substantially higher loadings. Overall, the three approaches yield nearly identical predictions of relative permeability, with the Maxwell equation exhibiting larger deviations only at elevated filler fractions. Figure 6b presents analogous results for MMMs containing cubic fillers. In this case as well, the electrical-circuit analogy and the finite-difference simulations produce indistinguishable results across a wide range of filler volume fractions. The Maxwell equation also provides an excellent approximation of the relative permeability for these systems.

(22) $$\frac{P_{eff}}{P_{p}}=\frac{P_{F}+2P_{p}-2\phi (P_{p}-P_{F})}{P_{F}+2P_{p}+\phi (P_{p}-P_{F})}$$

In gas permeation experiments, the time-lag method [25] is commonly employed to determine membrane diffusivity by monitoring the transient permeation rate of a gas through a non-porous membrane. As in the simulations conducted in this study, the upstream side of the membrane is subjected to a sudden pressure increase, while the downstream side is maintained under vacuum or near-vacuum conditions. As permeation proceeds, the pressure rise in the downstream chamber is recorded. Under steady-state permeation, the linear portion of the downstream pressure–time curve is extrapolated to the time axis; the resulting intercept is defined as the downstream time lag (*θ_d_*). The effective diffusion coefficient (*D_eff_*) can be calculated using Equation (23). Advances in instrumentation now allow for precise measurement of the pressure decay in the upstream reservoir, enabling determination of the upstream time lag (*θ_u_*) and, consequently, the effective diffusivity via Equation (24) [26]. Estimates of the relative diffusivity obtained from the upstream and downstream time-lag analyses are presented in Figure 6 as a function of the filler volume fraction for MMMs containing spherical and cubic particles. For a neat polymer membrane (*ϕ* = 0), both time-lag methods yield diffusivity values identical to those used in the simulations. As the filler volume fraction increases, the relative diffusivity estimated using the downstream time lag increases linearly for MMMs with spherical fillers, whereas for cubic fillers it increases more modestly and exhibits a maximum near a volume fraction of approximately 0.30. In contrast, the relative diffusivity estimated from the upstream time lag decreases with increasing filler content. Thus, the two time-lag methods produce inconsistent diffusivity estimates for the same MMM, with trends that depend on particle geometry. These estimates are clearly incorrect. Previous studies have shown that accurate determination of the effective diffusivity in MMMs using the time-lag method requires a sufficiently large number of repeatable filler layers along the permeation direction [22,27]. While the permeability of an MMM can be predicted with high accuracy using a single repeatable elementary unit in the permeation direction, this is not the case for diffusivity estimation. A detailed examination of this discrepancy lies beyond the scope of the present work, whose primary objective is to demonstrate that the equivalent electrical-circuit approach can predict process variables with accuracy comparable to that of the finite-difference method.

(23) $$D_{eff}=\frac{L_{y}^{2}}{6\theta_{d}}$$

(24) $$D_{eff}=-\frac{L_{y}^{2}}{3\theta_{u}}$$

**Figure 7:**
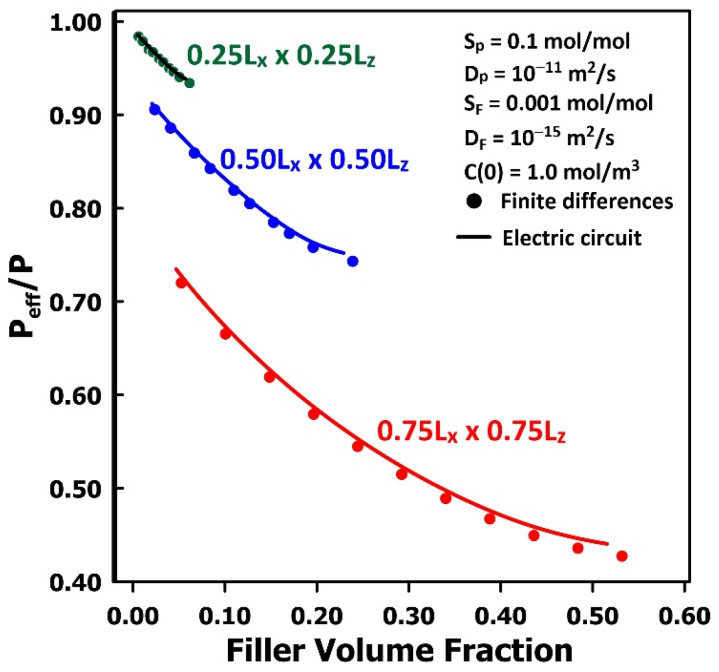
The comparison of the relative permeability of MMMs for three different filler particles as a function of the filler volume fraction, calculated with the equivalent electrical circuit and finite differences.

To further evaluate the capability of the equivalent electrical-circuit approach to solve Fick’s second law of diffusion for various filler geometries, a series of simulations was conducted for MMMs containing square-plate fillers of different thicknesses. The resulting estimates of relative permeability are presented in Figure 7 and compared with those obtained using the finite-difference method. The projected areas of the three square-plate fillers correspond to 12.5%, 25.0%, and 56.2% of the surface area of the repeatable elementary unit. The results demonstrate that both simulation methods yield nearly identical predictions of the relative permeability. Because the fillers possess a relatively low permeability, the relative permeability decreases with increasing filler volume fraction and projected area, consistent with the behaviour of barrier-type MMMs. Conversely, if the filler permeability exceeded that of the polymer matrix, the relative permeability would be greater than unity and would increase with filler loading.

**Figure 8:**
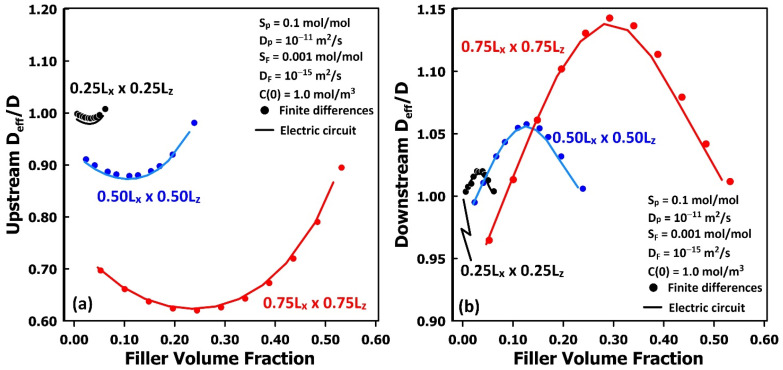
The comparison of the upstream (**a**) and downstream (**b**) relative diffusivity of MMMs as a function of the filler volume fraction, calculated with the equivalent electrical circuit and finite differences.

For each filler type shown in Figure 7, the relative diffusivity of a single repeatable elementary unit was evaluated using both the upstream and downstream time-lag methods for comparison. The relative diffusivities predicted using the equivalent electrical-circuit model, presented in Figure 8, are nearly identical to those obtained from the finite-difference method, demonstrating that the electrical network provides a robust framework for solving Fick’s second law of diffusion. The trends observed in the relative diffusivity depend on several factors, including the filler volume fraction, particle geometry, filler permeability, and the number of repeatable unit layers aligned along the permeation direction. The discrepancies revealed by the time-lag analyses for MMMs clearly warrant further investigation. Nonetheless, the present results confirm that the equivalent electrical-circuit approach offers an accurate and reliable means of modelling gas permeation through membranes.

**Figure 9:**
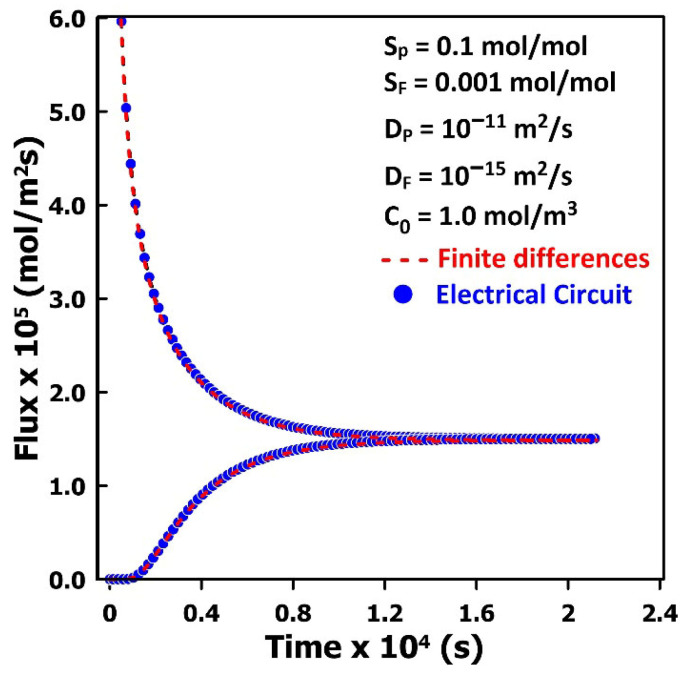
Comparison of the finite difference method and the electrical analogy for the estimation of the upstream and downstream fluxes for a MMM incorporating a nearly impermeable cuboid. Solutions were obtained for a 50 nm cubic elementary unit with a nanoparticle of dimensions 0.5*L_x_* × 0.92*L_y_* × 0.5*L_z_*.

The instantaneous inlet and outlet fluxes were computed using both simulation approaches for one of the MMM configurations presented in Figure 7 and Figure 8. In the electrical-circuit model, the fluxes were obtained directly from the average current density at the upstream and downstream faces of the repeatable elementary unit. In the finite-difference method, the fluxes were evaluated using Fick’s first law of diffusion based on the average concentration gradients at these same locations. As shown in Figure 9, both methods yield identical flux predictions, confirming the consistency of the two solution approaches.

### 3.3. Stability and Accuracy Evaluation

Through a series of numerical simulations, it was determined that the integration time step Δ*t* required to obtain a stable solution for the 3D electrical-circuit model is identical to the time step needed for solving Fick’s second law of diffusion using the finite difference method. For three-dimensional parabolic partial differential equations, such as the heat equation or Fick’s second law of diffusion, numerical stability is a critical consideration when employing finite-difference schemes. In explicit formulations, the time step Δ*t* must satisfy a stringent condition known as the Courant–Friedrichs–Lewy (CFL) condition to avoid instability [28]. Specifically, the time step must be sufficiently small in relation to the spatial grid sizes Δ*x*, Δ*y*, and Δ*z*. This criterion is expressed in Equation (25) for the three-dimensional solution [29]. In this investigation, the integration time step was chosen as 25% of the minimum value required for stability and was applied consistently across all simulations.

(25) $$\Delta t<\frac{1}{2D_{max}\left[\frac{1}{\Delta x^{2}}+\frac{1}{\Delta y^{2}}+\frac{1}{\Delta z^{2}}\right]}$$

In Equation (25), *D_max_* denotes the maximum diffusion coefficient within the computational domain. Exceeding this upper bound results in increasing numerical errors and the emergence of nonphysical solutions. In contrast, implicit schemes permit substantially larger time-step sizes and exhibit unconditional stability, albeit with a corresponding increase in computational cost.

**Figure 10:**
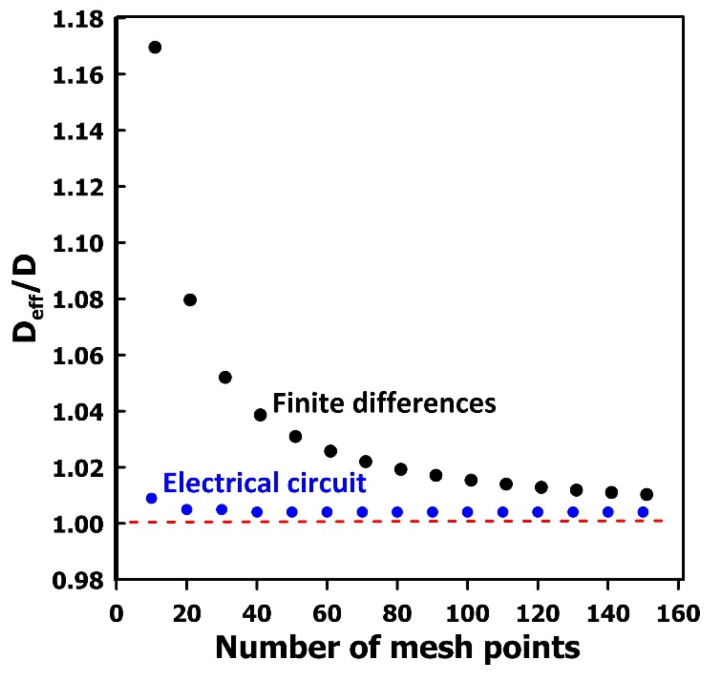
Comparison of the estimated diffusivity of a neat membrane as a function of the number of mesh points. The diffusivity values are obtained using the upstream time-lag method implemented through both the electrical-circuit and finite-difference formulations. The red line indicates the expected diffusivity ratio.

In addition to numerical stability, the spatial discretization of the computational domain plays a critical role in achieving accurate solutions. It is therefore standard practice to perform simulations at multiple discretization levels to verify that the results are independent of the mesh resolution. Figure 10 presents the estimated relative diffusivity of a neat membrane, obtained using the upstream time-lag method, as a function of the number of mesh points in the y-direction. When the electrical network method is employed, the prediction errors remain very small even with only 10 mesh points, and the results become effectively mesh-independent at approximately 20 mesh points (error < 0.4%). In contrast, the finite-difference method exhibits substantial prediction errors, reaching 17% for a 10-point mesh. The reduced accuracy of the finite-difference approach arises primarily from the evaluation of the concentration gradient at the upstream surface, particularly at the onset of the experiment. This gradient is required to compute both the flux and the pressure decay in the upstream reservoir. Any inaccuracy in its estimation shifts the pressure-decay curve and consequently leads to erroneous values of the upstream diffusivity. The equivalent electrical-circuit formulation avoids this limitation because the input flux (represented as an input current) is obtained directly, without the need for numerical differentiation. Although refining the mesh near the upstream boundary could improve gradient accuracy, this would increase computational cost. For the downstream time-lag method, the finite-difference approach yields negligible prediction errors even at relatively coarse mesh resolutions, owing to the smooth and consistently low concentration gradients in that region.

**Figure 11:**
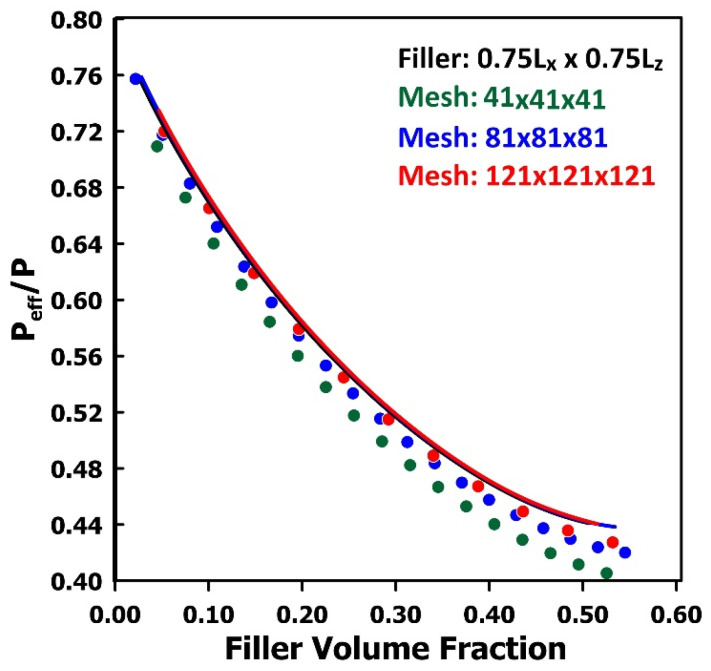
Comparison of the relative permeability of the MMM shown in Figure 7 as a function of filler volume fraction, computed using the equivalent electrical-circuit method and the finite-difference method at various discretization levels. Lines are for the electrical network, and symbols are for finite differences.

A series of simulations was conducted to evaluate the influence of mesh resolution on the estimated effective permeability of MMMs. The configuration corresponding to the larger square plate shown in Figure 7 was analyzed using three mesh densities, 41, 81, and 121 points in each spatial direction, while maintaining a constant filler-plate proportion (0.75*L_x_* × 0.75*L_z_*) and varying the filler thickness. The resulting relative permeabilities, computed as a function of filler volume fraction using both the electrical-circuit and the finite-difference methods, are presented in Figure 11. The three curves, obtained with the electrical-circuit method, are perfectly superimposed, indicating that the predicted relative permeability is effectively independent of the discretization level over the range of mesh sizes examined. In contrast, the finite-difference method yields three distinct permeability curves. As the mesh is refined, the finite-difference predictions progressively converge toward those obtained with the electrical-circuit method. It is therefore reasonable to hypothesize that, in the limit of sufficiently fine discretization, the finite-difference method would produce results identical to those of the electrical-circuit formulation. It should be noted that closer agreement between the two methods was observed for the smaller square-plate geometries, as well as for the spherical and cubic filler particles. Overall, the reduced sensitivity of the electrical-circuit network to mesh discretization strongly supports its use for the class of problems investigated here, as it provides accurate predictions while requiring substantially fewer computational resources.

### 3.4. General Discussion

There are several advantages to employing electrical analogies for the solution of unsteady-state heat and mass transfer problems:

(a) Owing to their conceptual simplicity, electrical circuits are often easier to analyze and implement computationally. Engineers can directly apply well-established electrical principles, such as Ohm’s and Kirchhoff’s laws, which map naturally onto the governing relations of heat and mass transfer.
(b) The governing equations associated with Fick’s second law of diffusion are straightforward to implement within an electrical-circuit framework, and considerably simpler than those required for finite-difference or finite-element formulations. Moreover, robust circuit-analysis tools and software can be readily adapted for efficient solution of heat and mass transfer problems. In this study, each unit cell was solved recursively using an explicit time-dependent formulation, analogous to a finite-difference approach, until a steady state was reached.
(c) The method provides strong conceptual clarity by establishing intuitive analogies: electrical resistance corresponds to mass or thermal resistance, voltage to concentration or temperature difference, and electrical current to mass or heat flux.
(d) Because many heat and mass transfer problems exhibit nonlinear behaviour, electrical analogies can be adapted to represent such effects accurately. This was demonstrated in the present investigation of MMMs, where distinct diffusivity and solubility coefficients for the polymer and filler phases were represented through different resistances and capacitances.
(e) Since the resistance and capacitance of each discrete three-dimensional volume can be specified independently, the approach readily accommodates nonlinear or anisotropic transport properties. For example, a capacitance element could be formulated to follow a Langmuir isotherm. As a result, the electrical circuit model, developed here for rubbery polymers, can be extended to represent the permeation of glassy polymers.
(f) The electrical analogy enables direct and accurate computation of upstream and downstream fluxes. In contrast, the finite-difference method requires flux evaluation from concentration gradients at the membrane surfaces, which may introduce significant numerical error.
(g) The equivalent electrical-circuit formulation exhibits reduced sensitivity to mesh discretization, allowing for accurate solutions to be obtained with fewer mesh points and, consequently, lower computational cost.
(h) The electrical circuit model, similar to other transport models, can be used to determine membrane transport parameters by fitting the pressure (or concentration) transients obtained from time-lag gas permeation experiments.

## 4. Conclusions

This study demonstrates the effectiveness of using an equivalent electrical network to model the transient diffusion of gases through non-porous membranes. By recasting Fick’s second law of diffusion within an electrical-circuit framework, we introduce a novel yet intuitive methodology that facilitates the analysis of mass-transfer processes, particularly in complex mixed-matrix and multilayered membranes. The analogy between electrical conduction and molecular diffusion not only provides conceptual clarity but also enables the use of well-established circuit analysis techniques to efficiently solve time-dependent transport problems.

While electrical analogies have long been employed to illustrate mostly steady-state behaviour in heat and mass transfer, our extension to dynamic systems underscores their broader applicability and relevance. This approach opens new avenues for both theoretical modelling and experimental design, especially in cases where direct measurement of transport parameters is difficult or impractical. Moreover, it reinforces the pedagogical value of analogical reasoning in engineering education, bridging abstract mathematical formulations with tangible physical insights.

Future work may explore the integration of this method with digital simulation tools, the modelling of reactive or multi-component systems, and the development of experimental setups that physically embody the proposed electrical analogies. Ultimately, the enduring utility of electrical analogies affirms their place not just in the history of engineering but in its evolving toolkit for solving complex transport phenomena.

## Figures and Tables

**Table 1:** Equivalences between heat transfer, mass transfer, and electrical circuit.

Variables	Heat Transfer	Mass Transfer	Electrical Circuit
Potential	$q=Ak\frac{\Delta T}{\Delta y}\left(\frac{J}{s}\right)$	$J=ADS\frac{\Delta C}{\Delta y}=AP\frac{\Delta C}{\Delta y}\left(\frac{mol}{s}\right)$	$I=\frac{\Delta V}{R}\;\left(\frac{V}{\Omega }\;or\;Ampere\right)$
Flux	$Acc=A\Delta y\rho C_{P}(T-T_{ref})\left(J\right)$	$Acc=A\Delta ySC\left(mol\right)$	$Acc=\mathrm{Charge}=Q=CV\left(C\right)$
Capacitance	C=AΔyρCP (JCo)	$C=A\Delta yS\;(m^{3})$	*C*
Resistance	R=ΔyAkCo sJ	$R=\frac{\Delta y}{ADS}=\frac{\Delta y}{AP}\left(\frac{s}{m^{3}}\right)$	*R*

## Data Availability

Data are available upon request to the corresponding author.

## Nomenclature

Main variables			
*A*	Surface area: *L_x_L_z_* or Δ*x*Δ*z* (m^2^)	*Q*	Electrical charge (C)
*C*	Concentration (mol/m^3^)	*R*	Resistance (Ω)
*C*	Capacitance (F)	*R*	Ideal gas constant (8.314 J/mol-K)
*C_0_*	Upstream concentration (mol/m^3^)	*S*	Solubility
*D*	Diffusivity (m^2^/s)	*t*	Time (s)
*I*	Current (A)	*U*	Center voltage of a unit cell (V)
*L*	Dimension (m)	*V*	Interface voltage of a unit cell (V)
*N*	Number of layers or discrete volumes	*x*	*x*-coordinate
*p*	Pressure (kPa)	*y*	*y*-coordinate
*P*	Permeability (m^2^/s)	*z*	*z*-coordinate
Greek symbols			
Δ	Space or time increment	*θ*	Time lag (s)
*ϕ*	Filler volume fraction		
Subscripts/Superscripts			
*C*	Capacitance	*P*	Polymer
*d*	Downstream	*t*	time
*F*	Filler	*u*	Upstream
*i*	Index of *x*-axis [1 to *N_x_*]	*x*	*x*-coordinate
*j*	Index of *y*-axis [1 to *N_y_*]	*y*	*y*-coordinate
*k*	Index of *z*-axis [1 to *N_z_*]	*z*	*z*-coordinate

## Appendix A. Sources of Membrane and Filler Transport Properties

Accurate simulation of gas permeation through both neat and MMMs requires reliable values for the diffusivity and solubility of the polymer phase and, for mixed-matrix systems, of the dispersed filler phase as well. This appendix provides a concise overview of several experimental and computational approaches commonly used to determine these parameters.

### Appendix A.1. Polymer Phase Parameters

For rubbery polymers, the diffusivity (*D_P_*) and solubility (*S_P_*) of the polymeric matrix are typically treated as intrinsic properties of the neat polymer and are obtained experimentally. These parameters, together with the permeability (*P*), are commonly obtained from constant-volume/variable-pressure time-lag measurements. The gas diffusivity in the polymer is derived from the downstream time lag ($D_{P}=L_{y}^{2}/6\theta_{d}$), while the steady-state slope of the downstream pressure rise provides the permeability. *D_P_* can also be determined from the upstream time lag ($D_{P}=-L_{y}^{2}/3\theta_{u}$) and *P* from the steady-state slope of the upstream pressure decay, when the constant-volume system includes this option [26]. The solubility is then calculated as the ratio of permeability to diffusivity $\left(S_{P}=P/D_{P}\right)$. Gas solubility may also be measured directly through gas-uptake experiments, such as gravimetric or volumetric sorption.

For glassy polymers, nonlinear sorption behaviour is often described using the dual-mode sorption model, which yields Henry’s law and Langmuir parameters (hole affinity constant and hole capacity constant) that can be used to define an effective solubility. In practice, for membrane transport simulations, *D_P_* and *S_P_* obtained from time-lag permeation experiments, and, when available, complementary sorption data, are treated as fixed inputs to the MMM model. These transport properties for glassy polymers are, in reality, effective, not intrinsic parameters.

### Appendix A.2. Filler Phase Parameters

The approach used to determine the transport properties of a filler phase depends strongly on whether the material is crystalline or porous (e.g., zeolites, metal–organic frameworks) or non-porous and essentially inert (e.g., silica, glassy inorganic oxides). For porous or crystalline fillers, the diffusivity (*D_F_*) can be estimated from the gas uptake kinetics, typically measured through time-resolved gravimetric or volumetric sorption experiments. Fitting appropriate Fickian diffusion models to the transient uptake curves yields *D_F_*. The solubility (*S_F_*) is obtained from the equilibrium sorption isotherm of the gas in the filler. Because sorption in microporous materials is often nonlinear, commonly described by Langmuir or dual-site Langmuir models, the resulting *S_F_* may be pressure-dependent and must be represented using an appropriate isotherm formulation, which can be easily incorporated in the simulation program. In many cases, literature data for a given gas–filler pair provide suitable estimates of both diffusivity and sorption behaviour. For materials such as MOFs and zeolites, molecular simulations (e.g., molecular dynamics or Monte Carlo methods) and structure–property correlations can also be used to approximate *D_F_* and *S_F_*.

If the filler is effectively impermeable to the penetrant gas, its solubility and diffusivity can be set to zero, and the filler is treated as a barrier phase. Under these conditions, the filler influences the MMM primarily through geometric and interfacial effects, such as tortuosity, percolation, or interfacial voids, rather than through intrinsic parameters.
